# Effect of alkaline preswelling on the structure of lignins from *Eucalyptus*

**DOI:** 10.1038/srep45752

**Published:** 2017-05-02

**Authors:** Wei-Jing Chen, Sheng Yang, Yun Zhang, Yun-Yan Wang, Tong-Qi Yuan, Run-Cang Sun

**Affiliations:** 1 Beijing Key Laboratory of Lignocellulosic Chemistry, Beijing Forestry University, Beijing, 100083, PR China; 2 Department of Bioproducts and Biosystems Engineering, University of Minnesota, Saint Paul, Minnesota 55108-6130, United States

## Abstract

A clear elucidation of structural feature of whole lignin in plant cell wall is of great importance for understanding lignin biosynthesis mechanism and developing lignin based chemicals or materials under the current biorefinery scenario. Swollen residual enzyme lignin (SREL) has been identified as an ideal representative for native lignin in the plant walls. To investigate the influence of preswelling conditions on the structural features, the SREL obtained through preswelling the ball-milled *Eucalyptus* wood powder in 2, 4 and 8% NaOH solutions and subsequent *in-situ* enzymatic hydrolysis were thoroughly characterized. A cellulolytic enzyme lignin (CEL) was also prepared as a comparison. The quantitative NMR analyses indicated that the relative contents of *β-O*-4′ linkages in SRELs were higher than that in CEL. The lignin structure tended to undergo more destruction with the elevated NaOH concentration. A relatively low NaOH concentration (2% in this study), which could be applied to effectively remove hemicelluloses and transform cellulose structure from cellulose I to cellulose II, was competent to prepare SREL as an ideal representative for the protolignin. An optimization of SREL preparation was essential for a better understanding of the whole protolignin.

A clear elucidation of the structural features of protolignin in plant cell wall is of great importance for understanding lignin biosynthesis mechanism and developing lignin-based chemicals or materials under the current lignocellulosic biorefinery scenario^1^. For a long time, milled-wood lignin (MWL) and cellulolytic enzyme lignin (CEL) have been considered as ideal representative sources of protolignin^2^,^3^,^4^. Some modified methods to prepare CEL were also subsequently brought forward, such as the isolation of milled-wood lignin involving solvent swelling prior to enzyme treatment as described by Chen *et al*.^5^ and Zhang *et al*.^6^, and the enzymatic hydrolysis lignin isolation proposed by Wu and Argyropoulos^7^. All these methods have made great contributions to the structural elucidation of lignin in plant cell wall. However, these time-consuming procedures usually produce poor yields of lignin and incomplete information about lignin macromolecular structures due to degradation or modification during isolation processes. For these reasons, the panoramic structural properties of whole protolignin in plant cell wall can hardly be unveiled through the foregoing methods.

To adequately protect the original structural features of lignin in plant cell wall, an *in-situ* characterization method by using high-field solution-state NMR (i.e. whole cell wall dissolution systems) was proposed^8^,^9^,^10^. The modified (acetylated) or untreated ball-milled plant cell walls can be dissolved in deuterated solvents (single or mixed systems, such as dimethylsulfoxide (DMSO)-*d*_6_ or DMSO-*d*_6_/pyridine-*d*_5_ systems), and the mixtures can be analyzed by two-dimensional heteronuclear single quantum coherence (2D-HSQC) to elucidate the whole lignin structure. The solvent mixture of DMSO-*d*_6_ containing 1-ethyl-3-methylimidazolium acetate ([Emim]OAc-*d*_14_) proposed by Cheng *et al*.^11^ was also able to dissolve lignocellulosic material completely, and subsequently a 2D HSQC NMR spectrum of the entire array of plant cell wall polymers could be obtained by using such a solvent system. However, it should be highlighted that the abundant carbohydrates in plant cell wall severely impede the identification of the lignin cross-signals and the quantification of the identified lignin structures^9^,^10^. In addition, the high cost deuterated ionic liquids ([Emim]OAc-*d*_14_), which were used to dissolve the ball milled plant cell wall as co-solvents, restricted the application and extension of this method. From the viewpoint of structural analysis, a simple and economical isolation process, which is able to separate lignin and carbohydrates efficiently with minimal modification on lignin macromolecular structures, will facilitate a better understanding of whole protolignin. Therefore, an ideal representative for native lignin should be isolated as much of the lignin as possible by a simple and economical process, while minimizing the extent of chemical modification.

To achieve the above-mentioned purposes, a new paradigm of lignin isolation method was proposed for the first time by our group^1^. A swollen residual enzyme lignin (SREL) as a residual lignin isolated from ball-milled plant cell wall, instead of dioxane-extracted lignin, was obtained by preswelling under a mild alkaline condition (4% NaOH, 25 °C, 24 h) followed by *in-situ* enzymatic hydrolysis to remove carbohydrates as much as possible. The extremely high yield (95%), low carbohydrate content and ideal solubility in DMSO-*d*_6_ make SREL an ideal candidate for the whole lignin structural analysis. However, it should be noted that the alkaline preswelling treatment could alter the structures of protolignin in plant cell wall to some extent^1^. It is believed that the differences in lignin structural features among different SRELs may be caused by the variation in preswelling condition (especially for the different concentrations of NaOH). This will significantly influence the understanding of the whole protolignin structure in plant cell wall. However, the investigation about this scientific issue was rare, and the impact mechanism was also not clear. Therefore, a study on the structural features of SRELs obtained from different preswelling conditions (different NaOH concentrations) is essential and valuable in the field of lignin chemistry.

In this study, the SRELs obtained under different preswelling conditions (different NaOH concentrations) followed by *in-situ* enzymatic hydrolysis were evaluated by comparing the yields, compositions and structural features. A CEL sample isolated under the same enzymatic hydrolysis condition as employed in SREL preparation was used as a comparison. The quantitative information, including functional groups, syringyl/guaiacyl (S/G) ratio and major substructures (*β-O*-4′, *β-β*′ and *β-*5′), were obtained by using ^31^P and 2D-HSQC NMR techniques.

## Results and Discussion

### Characterization of the preswelled plant cell walls

**Figure 1:**
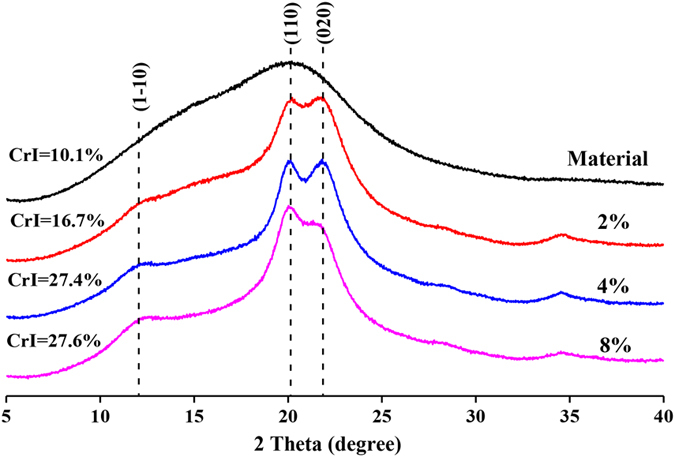
X-ray diffractograms of ball-milled *Eucalyptus* plant cell wall and ball-milled *Eucalyptus* plant cell wall preswelled in 2%, 4% and 8% sodium hydroxide solution.

The impact of NaOH concentrations employed in preswelling treatement on the chemical compositions of the ball-milled extractive-free *Eucalyptus* wood are listed in Table 1. By comparing with the composition of the raw material (ball-milled plant cell wall without preswelling), it can be found that the relative contents of the hemicelluloses in the preswelled plant cell walls significantly decreased, meanwhile the relative contents of cellulose in these samples obviously increased. This indicated that a large proportion of hemicelluloses were removed during the preswelling processes, and the compact structure of plant cell wall was disrupted simultaneously, which was significantly beneficial for the separation of the remained lignin and cellulose in the preswelled plant cell wall. The *CrI* values (Fig. 1) of the ball-milled plant cell wall and the samples preswelled with 2, 4 and 8% NaOH solutions were estimated to be 10.1, 16.7, 27.4 and 27.6%, respectively. The increase of *CrI* value after preswelling may be mainly caused by the removing of amorphous hemicelluloses and the inevitable loss of lignin fractions. It was also found that all the preswelled plant cell wall samples exhibited representative Miller indices for the reflections (1–10), (110) and (020) for cellulose II at 12.8°, 20.2° and 22.0°, repectively^12^. It is a typical XRD pattern of the transform state from cellulose I to cellulose II as a result of the cellulose I lattice expanding during the alkaline preswelling processes under different NaOH concentrations^13^. The board peak at around 16° (consisted with two peaks at 15.8° and 16.4°), which was assigned to typical diffraction patterns of cellulose I, could be found in the XRD curve of the raw material. However, it was very weak in the XRD curve of the 2% aqueous NaOH treated plant cell wall, and gradually disappeared when NaOH concentration was increased from 4% to 8%. Along with the foregoing phenomenon, it could be observed that the decrease in the intensity of the peak at 22.0° was accompanied by the increasing intensity at 20.2° with the elevated NaOH concentration. The increase in NaOH concentration boosted the crystal form transformation of cellulose was boosted, and subsequently promoted the separation of the remaining lignin and cellulose in the plant cell wall through enzymatic hydrolysis^14^,^15^. In addition, although the relative content of cellulose in the preswelled samples increased after alkaline pretreatment, its absolute quality was decreasing, which was caused by the peeling reaction of terminal glucose in alkaline conditions^16^,^17^.

**Figure 2:**
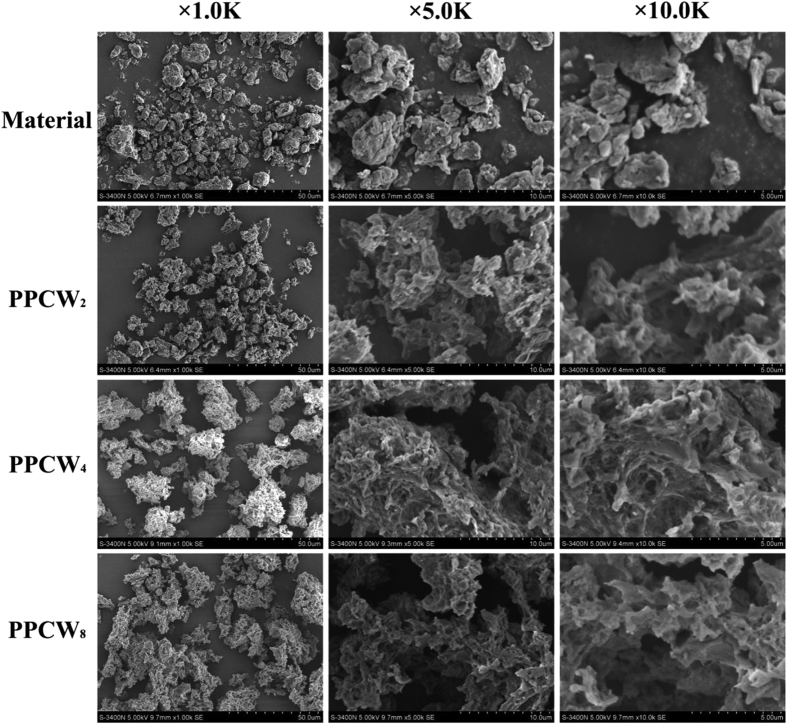
SEM photomicrographs of ball-milled *Eucalyptus* plant cell wall and ball-milled Eucalyptus plant cell wall preswelled in 2%, 4% and 8% sodium hydroxide solution. (PPCW means the preswelled plant cell wall).

The SEM images (Fig. 2) revealed that the untreated ball-milled plant cell wall had a rigid and compact morphology, whereas the surface morphology of the preswelled samples exhibited a rough and loosened structure, and the roughness increased with the elevated NaOH concentration. Nevertheless, the relative contents of lignin fraction in the preswelled samples were similar with one another. The differences in the relative contents of cellulose and hemicelluloses in the preswelled samples obtained after preswelling under different NaOH concentrations (2, 4 and 8%), were also not obvious (Table 1). All of these results implied that part of lignin was dissolved and removed during the preswelling process. It should be noted that in SREL preparation, part of the dissolved lignin fraction could regenerate during the acidification process before enzymatic hydrolysis step. This will impede the degradation of cellulose and hemicellulose in the plant cell wall^18^.

### Composition of the lignin samples

All the SREL samples were prepared through preswelling of plant cell walls followed by *in-situ* enzymatic hydrolysis, and a CEL sample was prepared as a comparison. The yields and sugar compositions of the SRELs and CEL are listed in Table 2. The results showed that the pure lignin yields of SREL_2_, SREL_4_ and SREL_8_ were 90.6, 91.1 and 90.6%, respectively, based on the Klason lignin of the ball milled plant cell wall, which were considerably higher than the yield of CEL (20.3%). Therefore, all the SRELs prepared in this study were more appropriate representatives for the whole protolignin than CEL. Although lignin yields of SRELs were substantial, some sugars were still found in the samples. It was observed that the glucose contents in SREL_2_, SREL_4_ and SREL_2_ were 1.60%, 1.75% and 1.56%, respectively. The glucose was the major sugar in all the SREL samples followed by xylose. However, the content of xylose was the highest in CEL (1.41%) followed by glucose (1.12%). These differences between the associated sugars should be caused by the different conditions applied in the isolations of SREL and CEL. The higher content of glucose in SREL should be attributed to the small amount of obstinate cellulose after the enzymatic hydrolysis, while the abundant xylose in CEL was related to the potential lignin–carbohydrate complex (LCC). This phenomenon was well in agreement with our previous study^19^. All three SREL samples could be dissolved in DMSO-*d*_6_ even in the presence of small amounts of sugars. It should be mentioned that the SREL_0.5_ and SREL_1_ could also be obtained from 0.5 and 1.0% NaOH solution preswelling treated plant cell walls. However, their solubility in DMSO was poor (as shown in Fig. S1), and the corresponding structural information of lignin could not be studied by NMR analyses. Therefore, the corresponding data about these two lignin samples was not provided in the present study.

The extremely similar pure lignin yields of the three SREL samples (SREL_2_, SREL_4_ and SREL_8_) indicated that the NaOH concentration used in this study have no obvious effect on the preparation of SREL. However, it should be highlighted that the macromolecular structure of protolignin could, to some extent, be modified under alkaline conditions and some important information may be lost. For these reasons, the structural features of the SREL samples obtained under different preswelling conditions should be carefully investigated to find an ideal sample for the whole protolignin study.

### 2D-HSQC spectra analysis

**Figure 3:**
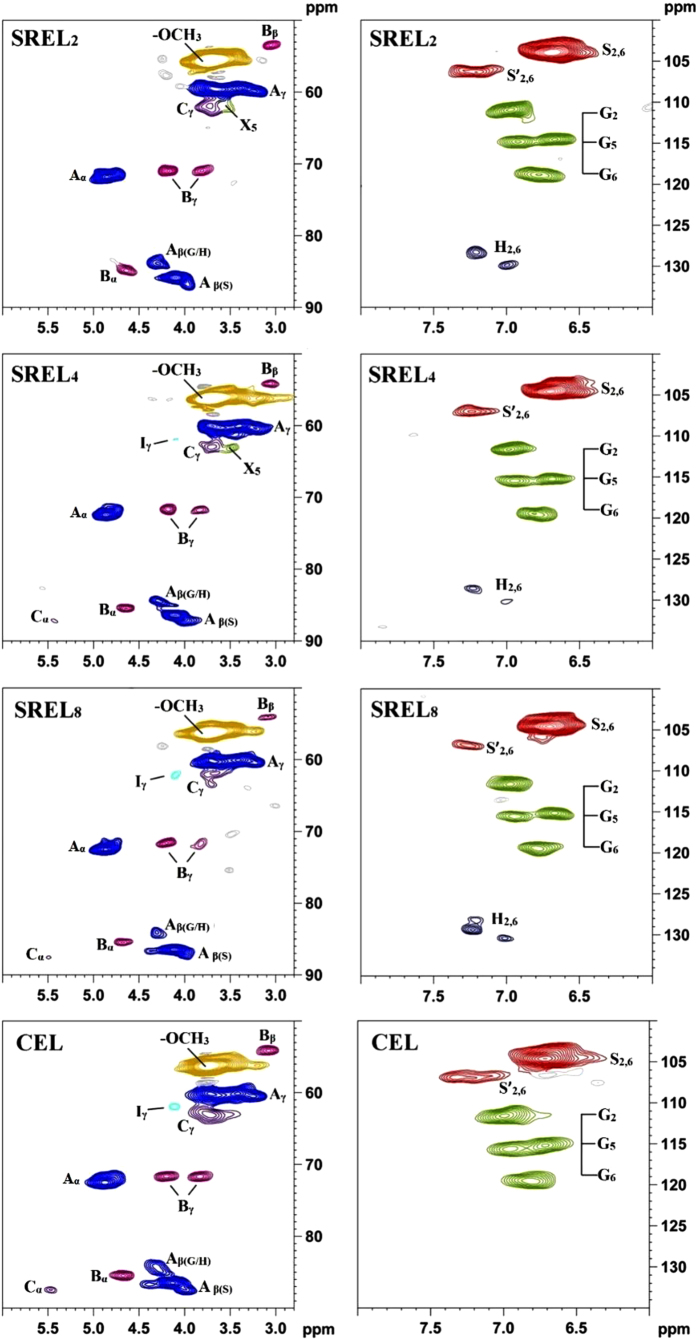
Side-chain and aromatic region in the 2D HSQC NMR spectra of different lignin samples.

**Figure 4:**
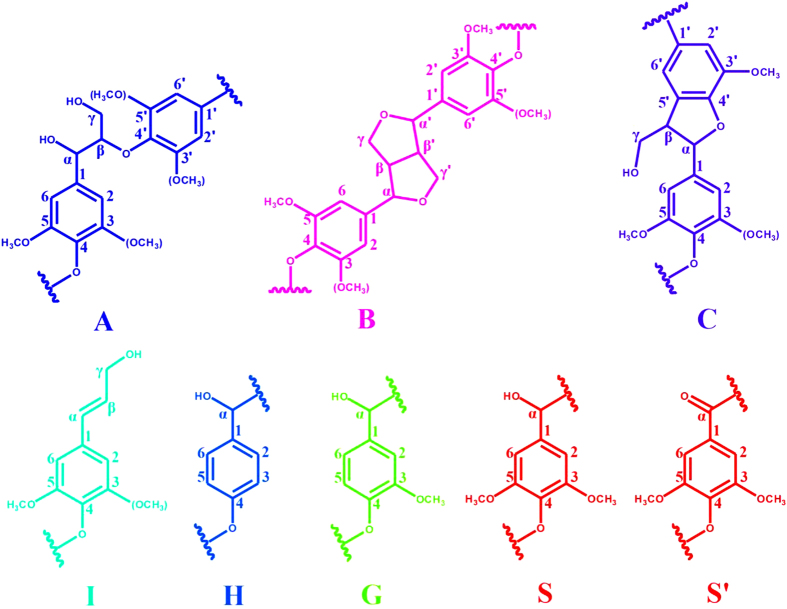
Key structural details of different lignin samples: (**A**) *β-O*-4′ aryl ether linkages with a free -OH at the *γ*-carbon; (**B**) resinol substructures formed by *β-β′, α-O-γ′*, and *γ-O-α′* linkages; (**C**) phenylcoumaran substructures formed by *β*-5′ and *α-O*-4′ linkages; (I) *p*-hydroxycinnamyl alcohol end groups; (**H**) p-hydroxyphenyl units; (**G**) guaiacyl units; (S) syringyl units; (S′) oxidized syringyl units with a C_*α*_ ketone; (X) *β*-D-Xylp.

The detailed chemical structures of the lignin samples (SREL_2_, SREL_4_, SREL_8_ and CEL) were investigated by using the 2D HSQC NMR technique in this study. The side-chain and aromatic regions of the 2D HSQC spectra of all the lignin samples are shown in Fig. 3, and the main substructures are displayed in Fig. 4.

In the side-chain regions of the spectra of all the lignin samples, the substructures, such as *β-O*-4′ aryl ethers (A), resinols (B), phenylcoumarans (C), could be easily assigned according to the previous publications^20^,^21^,^22^,^23^. All the spectra showed prominent cross-signals corresponding to *β-O*-4′ linkages. The C_α_-H_α_ correlations of the *β-O*-4′ linkages were observed at *δ*_C_/*δ*_H_ 72.0/4.72, while the C_*β*_-H_*β*_ correlations were seen at *δ*_C_/*δ*_H_ 84.0/4.31 and 86.0/4.12 for the substructures linked to the G/H and S units, respectively. The C_*γ*_-H_*γ*_ correlations in the *β-O*-4′ substructures were found at *δ*_C_/*δ*_H_ 60.1/3.40–3.73, and partially overlapped with the cross-signals of C_5_-H_5_ of xylans as shown in the spectra of SREL_2_ and SREL_4_. Excepting for the abundant *β-O*-4′ linkages, resinol also appeared in the spectra of all the lignin samples in noticeable amounts as indicated by their C_*α*_-H_*α*_, C_*β*_-H_*β*_ and the double C_*γ*_-H_*γ*_ correlations at *δ*_C_/*δ*_H_ 84.8/4.66, 53.5/3.07, 71.2/3.82 and 4.18, respectively. Phenylcoumaran was also detected in a minor amount. Moreover, the cross-signals located at *δ*_C_/*δ*_H_ 62.0/4.11, which is assigned to the C_*γ*_-H_*γ*_ correlation of *p*-hydroxycinnamyl alcohol end groups (I), was also observed in the spectra of different lignin samples. The cross-signals assigned to C_*γ*_-H_*γ*_ of *p*-hydroxycinnamyl alcohol end groups and C_*α*_-H_*α*_ of phenylcoumaran were not found in the spectrum of SREL_2_. However, these two cross-signals could be observed at a lower contour level (not shown).

In the aromatic regions of the spectra of the SREL samples, the cross-signals of the syringyl (S), guaiacyl (G) and *p*-hydroxyphenyl (H) units could be clearly distinguished. The S units showed an obvious cross-signal for the C_2,6_-H_2,6_ correlations at *δ*_C_/*δ*_H_ 103.5/6.66, and the signal for the C_*α*_-oxidized S units (S′) was found at *δ*_C_/*δ*_H_ 106.3/7.32. In addition, three different cross-signals, which are assigned to G units, were observed at *δ*_C_/*δ*_H_ 110.8/6.97 (C_2_-H_2_), *δ*_C_/*δ*_H_ 114.5/6.70 and 115.1/6.95 (C_5_-H_5_), and *δ*_C_/*δ*_H_ 119.0/6.78 (C_6_-H_6_). The C_2,6_–H_2,6_ correlations in H unit appeared at *δ*_C_/*δ*_H_ 127.0/6.95. It could be found that the spectra of all the SREL samples were similar with one another, and were also almost identical to the 2D HSQC spectra of CEL. However, the cross-signal of H units observed in the spectra of SRELs disappeared in the 2D HSQC spectrum of CEL. According to a previous study, the cross-signal at *δ*_C_/*δ*_H_ 127.0/6.95 may also originate from residual cellulase^1^. Nevertheless, the absence of other cross-signals assigned to cellulase implied that the cross-signals appeared at this position in the spectra of the three SRELs in the present study were mainly originated from H units. This indicated that the information of H units in the protolignin could be obtained through SREL, and it was a more precious sample for the whole lignin study as compared with CEL.

To distinguish the differences in the structural features of the obtained lignin samples, a quantitative analysis is of great necessity. The S/G/H ratio and main linkages (referred to as a percentage of the total side chains) of the lignin samples, which were calculated from the 2D HSQC spectra based on the method described in a previous publication^19^, are shown in Table 3. It could be found that all the lignin samples displayed a predominance of the *β-O*-4′ aryl ether units followed by the *β-β*′ units. The relative contents of *β*-5′ linkages of all the lignin samples were significantly lower than those of the *β-O*-4′ and *β-β*′ linkages. It was observed that the relative content of the *β-O*-4′ linkages was lower in CEL than those of the same linkages in the SREL samples. This result was caused by the different isolation methods used. For CEL, most of the lignin fractions in the middle lamella (ML) and part of the lignin fractions in the secondary wall (S2) were isolated, and a large amount of lignin fractions in the S2 were discarded. It was reported that the S/G ratio in the ML was lower than that in the S2^24^. The relative low S unit proportion in lignin macromolecule leads to a low proportion of *β-O*-4′ aryl ether linkages^1^,^10^,^25^. The relatively low S/G ratio of CEL (2.3) also confirmed this result. In fact, the S/G ratio was an average value of lignin collected from different parts of plant cell wall. In the present study, the SREL samples were obtained as residues after preswelling followed by *in-situ* enzymatic hydrolysis. Almost all the lignin fractions in the plant cell wall were collected in the form of SREL. Thus, the high ratio of S units in the S2 leaded to a higher relative content of *β-O*-4′ linkages in the SREL samples. No obvious difference in the relative contents of *β-O*-4′ linkages could be found in the three SREL samples, and the contents of *β-β*′ linkages exhibited the same phenomenon. In addition, there was no significant difference of the S/G/H ratios among the three SREL samples. These implied that the preswelling conditions (different NaOH concentrations) used in this study had no significant effect on the lignin structure of the final SREL samples. As combined with the above results obtained in the analyses of the preswelled plant cell walls and composition lignin samples, it could be concluded that a relatively low NaOH concentration, which could effectively remove hemicelluloses and transform cellulose structure from cellulose I to cellulose II, was competent for preparing an ideal SREL sample for the whole protolignin study.

A comparison study of the SREL and the whole cell wall gel methods was of great necessity to confirm the reliability of this work. In this study, it was found that the content of *β-O*-4′ linkages in SREL samples was slightly higher than that in CEL sample. An analogous result has also been reported by a previous work^26^. In that work, the whole cell wall gel method was used to study the protolignin in *Eucalypt* wood, and the relative content of *β-O*-4′ linkages in the ball-milled plant cell wall sample was also slightly higher than that in the MWL sample. The relative content of *β-β*′ linkages (14.0–16.4%) of SREL samples in the present work was similar with that (14.0%) of the ball-milled plant cell walls obtained by the whole cell wall gel method^26^. In the case of aromatic region analysis of the lignin structure, the S/G ratios of the SREL samples were higher that of the CEL sample in this study, while an opposite result was reported by the previous literature^26^. This may be caused by the different solution state of the SREL and the ball milled plant cell wall samples in DMSO-*d*_6_. The presence of abundant polysaccharides hinders the dispersion of the ball-milled plant cell wall sample in DMSO-*d*_6_, and the quality of the obtained 2D HSQC spectrum was poor. In addition, the cross-signals of H units was not observed in the 2D HSQC spectrum of *Eucalypt* wood obtained through the whole cell wall gel method. This further confirmed the reliability of the SREL method for the whole protolignin study.

### ^31^P-NMR spectra analysis

**Figure 5:**
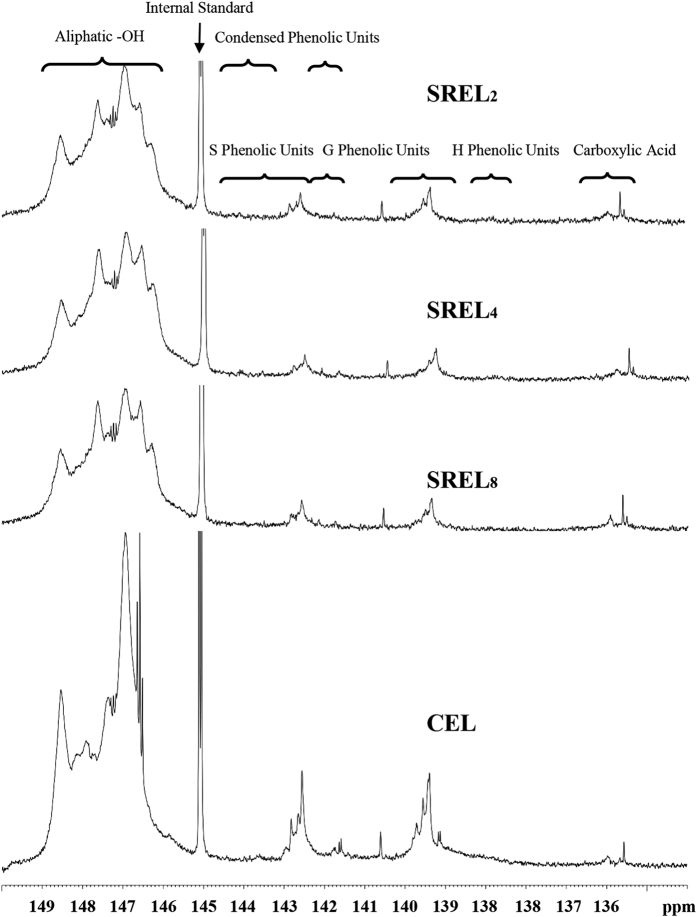
Quantitative ^31^P NMR spectra of different lignin samples.

To evaluate the functional groups of the lignin samples, quantitative ^31^P NMR technique was employed, and the ^31^P NMR spectra of all the lignin samples are shown in Fig. 5. The assignments and calculation methods were conducted according to previous publications^27^,^28^, and the corresponding results are listed in Table 4. It was observed that both of the aliphatic and phenolic -OH contents of the CEL were higher than the corresponding values of the SREL samples. Generally, the cleavage of *β*-*O*-4′ linkages will give rise to the aliphatic and phenolic hydroxyl groups^29^. Higher amount of aliphatic and phenolic -OH in the CEL than that in the SREL samples indicated that the CEL consisted of more degraded lignin fragments caused by ball milling. It also meant that some important structural information may be lost when CEL was used as a representative of the protolignin in plant cell wall. Lower amount of phenolic -OH in the SREL samples implied that the SREL contained more etherified lignin units^30^. The structural information provided by the SREL samples would be more representative of the protolignin structure. It was also found that with the increasing NaOH concentration, the content of aliphatic and phenolic hydroxyl groups in the obtained SREL samples increased gradually. This indicated that with the elevated NaOH concentration, more information of the structural features of the protolignin in plant cell wall was lost. Thus, with an appropriate preswelling condition and the sufficient subsequent enzymatic hydrolysis, the SREL_2_ should be the best lignin sample for the whole lignin study. In addition, non-condensed H-type phenolic -OH was not found in all of the lignin samples in the present study, indicating that the H units in plant cell wall should be mainly condensed-type. It could be concluded that a low NaOH concentration was preferred for ideal SREL preparation as long as the obtained PPCW could be fully hydrolyzed and the final lignin sample could be directly used to liquid NMR analysis for protolignin study.

## Materials and Methods

### Materials

Wood sample used in this study was obtained from the *Eucalyptus grandis* × *Eucalyptus urophylla*, a 5-year-old *Eucalyptus* tree harvested from Guangxi province, China. The wood sample was grinded and Soxhlet−extracted with toluene–ethanol (2:1, v/v) for 6 h. The extractive-free *Eucalyptus* wood meal was ball-milled in a planetary ball mill (Fritsch, Germany) according to a previous publication^31^. The cellulolytic enzyme (an enzyme cocktail containing cellulase and hemicellulase activities) used in this study was kindly supplied by Shanghai Youtell Biochemical Co., Ltd. The chemicals used in the present study were of analytical grade or better, and directly used as received.

### Preparation of SREL and CEL samplesN

The SREL samples were prepared according to the procedure described by Wen *et al*.^1^. The preswelling of the ball-milled plant cell wall were conducted in 2, 4 and 8% aqueous NaOH solutions, respectively. The subsequent *in-situ* enzymatic hydrolyses were carried out based on the approach reported by the previous publication^1^. The resulting lignin samples obtained as residues were labeled as SREL_2_, SREL_4_ and SREL_8_, respectively. In order to explore the influence of the NaOH concentration on the ball-milled plant cell wall during the preswelling process, three plant cell wall samples were preswelled with 2, 4 and 8% aqueous NaOH solutions, and then regenerated in water without the enzymatic process. The obtained preswelled plant cell wall samples were named as PPCW_2_, PPCW_4_ and PPCW_8_, respectively. The preparation of cellulolytic enzyme lignin (CEL) was performed under the same enzymatic hydrolysis condition that has been applied to SREL preparations as described above.

### Characterization of the PPCW samples

The compositions of the ball-milled and preswelled plant cell walls were analyzed according to the standard method recommended by National Renewable Energy Laboratory (NREL)^32^. All of the PPCW samples were characterized by X-ray diffraction (XRD) and scanning electron microscope (SEM). The XRD scans were recorded on a Bruker D8 Advance instrument (Bruker, Germany) ranging from 5 to 40° 2θ using a goniometer at a scanning speed of 2 °/min. The assay was conducted with Ni-filtered Cu Kα radiation (λ = 1.54 Å) at 40 kV and 40 mA. The crystallinity index (*CrI*) of the samples equals to the ratio of the area of the resolved crystalline peaks to the total area of a diffraction profile based on the obtained X-ray diffraction curves. The SEM analysis of these samples was performed on a Hitachi S-3400 N II (Hitachi, Japan) instrument at 15 kV. Before SEM observation, all samples were sputter-coated with gold to improve the conductivity and the quality of the SEM images. The images of all the samples were collected at magnifications of 1000, 5000 and 10000.

### Characterization of the lignin samples

The lignin yields and sugar compositions of the SRELs and CEL were also analyzed according to the NREL standard method^32^. The 2D HSQC spectra were recorded at 25 °C on a Bruker AVIII 400 MHz spectrometer (Bruker, Germany). For each sample, 60 mg of lignin was dissolved in 0.5 mL of DMSO-*d*_6_. A semi-quantitative method based on 2D HSQC spectra was used to calculate the relative amount of inter-linkages and S/G ratio of the lignin samples^19^. ^31^P NMR analysis was conducted based on the method reported by Granata and Argyropoulos^30^. ^31^P NMR spectra were acquired after the reaction of lignin with 2-chloro-4, 4, 5, 5-tetramethyl-1, 3, 2-dioxaphospholane (TMDP). The parameters used in quantitative ^31^P NMR experiment were listed as follows: 30° pulse angle, 2 s relaxation delay (*d*_1_), 64 K data points and 1024 scans. The analyses of the SREL and CEL samples were conducted three times, and the average value of the three repeated measurements was used as final result.

## Additional Information

**How to cite this article**: Chen, W.-J. *et al*. Effect of alkaline preswelling on the structure of lignins from *Eucalyptus. Sci. Rep.* **7**, 45752; doi: 10.1038/srep45752 (2017).

**Publisher's note:** Springer Nature remains neutral with regard to jurisdictional claims in published maps and institutional affiliations.

## Supplementary Material

Supplementary Figure S1

## Figures and Tables

**Table 1:** Compositional analysis of the raw material and preswelled plant cell walls.

Samples	Lignin (%)			Cellulose (%)	Hemicellulose (%)	Others (%)
	AIL^a^	ASL^b^	Total			
Material	29.07	5.74	34.81 (38.06)^c^	39.38 (43.05)	17.28 (18.89)	8.53
PPCW_2_	29.40	4.33	33.73 (37.67)	47.29 (52.82)	8.51 (9.51)	10.47
PPCW_4_	29.57	4.47	34.04 (39.60)	44.21 (51.44)	7.70 (8.96)	14.05
PPCW_8_	31.50	4.83	36.33 (37.86)	50.64 (52.77)	8.99 (9.37)	4.04

^a^AIL: acid-insoluble lignin.

^b^ASL: acid-soluble lignin.

^c^ Relative content (%) based on the weight sum of lignin, cellulose and hemicelluloses.

**Table 2:** Yield and carbohydrate contents of different lignin samples.

Samples	Yield (%)^a^	Carbohydrate content^b^ (%)							
		Rha	Ara	Gal	Glc	Man	Xyl	GlcA	GalA
SREL_2_	96.0(0.7)^e^ /90.6^d^	0.06 (0.01)	0.43 (0.11)	0.99 (0.25)	1.60 (0.22)	0.88 (0.30)	1.47 (0.17)	0.21 (0.08)	N.D^c^
SREL_4_	96.0(1.1) /91.1	0.08 (0.04)	0.28 (0.08)	0.79 (0.26)	1.75 (0.14)	0.68 (0.21)	1.17 (0.41)	0.38 (0.11)	N.D
SREL_8_	95.0(0.4) /90.6	0.04 (0.01)	0.35 (0.07)	0.77 (0.22)	1.56 (0.22)	0.69 (0.14)	1.15 (0.39)	0.07 (0.03)	N.D
CEL	20.3(1.1) /19.4	N.D	N.D	0.11 (0.02)	1.12 (0.13)	0.46 (0.17)	1.41 (0.13)	0.13 (0.04)	N.D

^a^Based on Klason lignin of the *Eucalyptus* wood.

^b^Rha = rhamnose, Ara = arabinose, Gal = galactose, Glc = glucose, Man = mannose, Xyl = xylose, GlcA = glucuronic acid, GlaA = galacturonic acid.

^c^Not detected.

^d^The yield without sugars.

^e^The value in the parenthesis is standard deviation.

**Table 3:** Quantification of the lignin fractions by a semi-quantitative 2D-HSQC method: results represented as percentage of total side chains.

Samples	*β-O*-4′^c^	*β-β*′	*β*-5′	S/G/H^a^
SREL_2_	83.1 (0.4)^b^	16.4 (0.3)	0.4 (0.3)	2.5:1.0:0.1
SREL_4_	85.2 (0.9)	14.0 (0.6)	0.8 (0.1)	2.9:1.0:0.1
SREL_8_	85.1 (0.9)	14.1 (0.4)	0.7 (0.3)	2.5:1.0:0.2
CEL	80.3 (1.5)	17.1 (0.9)	2.6 (0.6)	2.3:1.0:0.0

^a^S/G/H ratio obtained according to S/G/H = 0.5IS_2,6_/IG_2_/0.5IH_2,6_.

^b^The value in the parenthesis is standard deviation.

Note: The data in this table were calculated based on a semi-quantitative method.

**Table 4:** Functional groups of MWL determined by quantitative ^31^P-NMR method (millimole per gram).

Samples	Aliphatic OH	5-substituted phenolic -OH	Non-condensed Guaiacyl phenolic –OH	*p*-hydroxy phenyl OH	Carboxylic group
SREL_2_	0.208 (0.014)^b^	0.113 (0.034)	0.095 (0.022)	N.D^a^	0.006 (0.002)
SREL_4_	0.493 (0.023)	0.306 (0.022)	0.187 (0.031)	N.D	0.098 (0.025)
SREL_8_	0.555 (0.019)	0.359 (0.046)	0.196 (0.028)	N.D	0.120 (0.018)
CEL	0.934 (0.061)	0.515 (0.011)	0.419 (0.054)	N.D	0.068 (0.030)

^a^Not detected.

^b^The value in the parenthesis is standard deviation.
