# Supplementary material for: Effect of alkaline preswelling on the structure of lignins from Eucalyptus
Source: Sci Rep. 2017 May 2;7:45752. doi: 10.1038/srep45752 (PMC5411976; doi:10.1038/srep45752)
Supplement: Supplementary Figure S1 [file srep45752-s1.pdf]

Supporting information

Effect of alkaline preswelling on the structure of lignins from *Eucalyptus*

Wei-Jing Chen,[1,#] Sheng Yang,[1,#] Yun Zhang,[1] Yun-Yan Wang,[2] Tong-Qi Yuan,[1,*] Run-Cang Sun[1,*]

[1] Beijing Key Laboratory of Lignocellulosic Chemistry, Beijing Forestry University, Beijing, 100083, PR China

[2] Department of Bioproducts and Biosystems Engineering, University of Minnesota, Saint Paul, Minnesota 55108-6130, United States

*Corresponding author: Beijing Key Laboratory of Lignocellulosic Chemistry, Beijing Forestry University, Beijing, 100083, PR China. Tel./fax: +86-010-6233-6903; E-mail addresses: yangsheng230@bjfu.edu.cn (S Yang); ytq581234@bjfu.edu.cn (TQ Yuan); rcsun3@bjfu.edu.cn (RC Sun).

[#] Wei-Jing Chen and Sheng Yang contributed equally to this work.

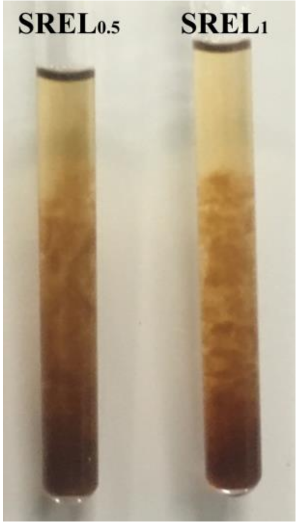

Figure S1. Image of $SREL_{0.5}$ and $SREL_1$ dissolved in DMSO-d_6 (photograph).
